# Recent advances and future challenges in nanosystems for ocular drug delivery

**DOI:** 10.1016/j.jpet.2025.103738

**Published:** 2025-12-30

**Authors:** Rami A. Shahror, Abdelrahman Y. Fouda

**Affiliations:** 1 Department of Pharmacology and Toxicology, College of Medicine, University of Arkansas for Medical Sciences, Little Rock, Arkansas; 2 Department of Clinical Pharmacy, Faculty of Pharmacy, Cairo University, Cairo, Egypt

**Keywords:** Nanosystems, Ocular diseases, Drug delivery

## Abstract

Ocular drug delivery faces significant challenges because of the eye’s complexity and anatomical barriers, such as the cornea, conjunctiva, and blood–retinal barrier, which limit drug penetration and bioavailability. Recent advances in nanotechnology-based drug delivery have led to the development of innovative delivery platforms, enabling targeted, sustained, and minimally invasive delivery for ocular diseases and injuries. This review outlines the recent advances in nanosystems-based ocular drug delivery and highlights the latest progress in targeting technologies based mainly on preclinical studies and selected clinical trial data. It covers a variety of nanosystems, including organic nanoparticles (NPs) such as liposomes, nanomicelles, nanosuspensions, nanoemulsions, dendrimers, and nanofibers. Additionally, it addresses inorganic NPs, which include gold NPs, silver NPs, silica NPs, and carbon nanotubes. Besides, we summarize the clinical challenges and regulatory aspects in nanotechnology-based ocular drug delivery. Finally, inspired by current advances and therapeutic strategies, we provide an insight into clinical applications and future research directions on nanosystems-based drug delivery. We highlight the need to overcome the challenges of using nanosystems in ocular drug delivery and fill the knowledge gap on their nanotoxicity and future development.

**Significance Statement:**

This review highlights recent advances in nanosystem applications for ocular drug delivery, summarizes up-to-date clinical trials utilizing nanosystems for ocular drug delivery, and discusses clinical challenges and directions for future development.

## Graphical abstract

**Figure:**
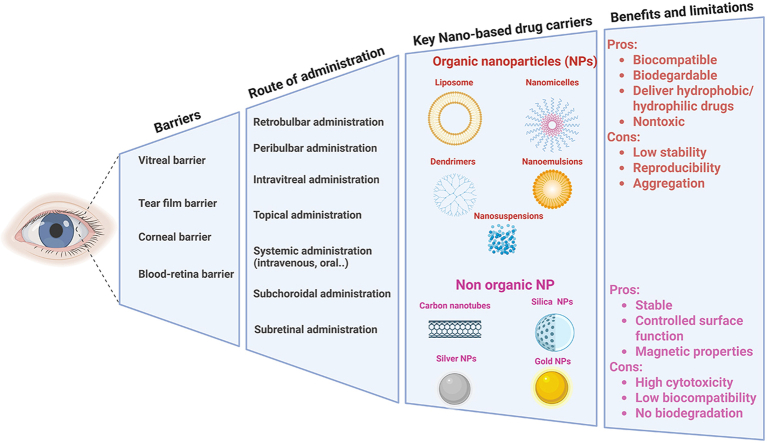

## 1 Introduction

The World Health Organization estimates that ocular diseases profoundly lead to vision impairment and significantly influence the quality of life of over 2.2 billion people globally.^1^ Nearly half of those affected by vision impairments have conditions that could be prevented or are not being treated. Additionally, vision impairment is linked to aging, diabetes, genetic conditions, and infections. The most prevalent blinding ocular diseases include cataracts, diabetic retinopathy (DR), age-related macular degeneration (AMD), and retinoblastoma. A recent study suggests that approximately 76 million people suffer from glaucoma, 196 million from AMD, and 92.6 million from DR worldwide.^2^ Despite the high prevalence of vision impairment, the effective delivery of pharmaceuticals to ocular tissues remains challenging because of the complexity of the eye and the anatomical barriers present in the eye’s structure.

**Fig. 1:**
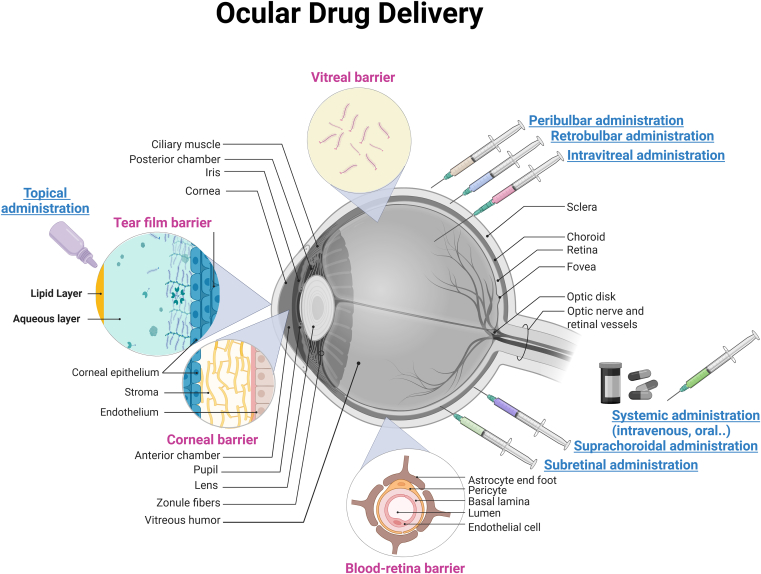
Ocular barriers and routes of drug administration. The eye’s ocular drug delivery barriers primarily include the tear film barrier, protects, and lubricates the surface of the eye via the innermost mucin layer; the corneal barrier, consists of the tight junctions in the corneal epithelial cells; the blood–retina barrier, mainly comprised from endothelial cells lining the retinal capillaries and glia cells, and the vitreal barrier, comprised from vitreous humor and solid fibrillar components. The most common routes of ocular drug administration are subretinal, suprachoroidal, transscleral, topical, peribulbar, retrobulbar, intravitreal, and systemic administration. Subretinal administration, performed in the operating room, is considered an effective route for delivering ocular drugs to the photoreceptor layer and retinal pigment epithelium. Suprachoroidal injection via a microneedle, a nonsurgical in-office procedure, delivers ocular therapeutics to the suprachoroidal space between the choroid and sclera. Transscleral administration is a minimally invasive method for delivering local, sustained ocular therapeutics to the posterior segment of the eye. Topical administration is used to deliver 90% of all ocular therapeutics, mainly to the anterior eye segments, usually via drops or a fine mist spray. Retrobulbar and peribulbar administration are mainly used for delivering anesthetic agents into retrobulbar and peribulbar blocks. Intravitreal injection, performed in an office setting, delivers ocular therapeutics to the vitreous humor. Systemic administration through oral or intravenous routes allows distribution of ocular therapeutics to the posterior segment of the eye.

The eye can be broadly classified into anterior and posterior segments.^3^ The anterior segment consists of the cornea, conjunctiva, iris, ciliary body, and lens. It is divided into 2 chambers, anterior and posterior, where the aqueous humor is produced in the posterior chamber. The posterior segment comprises the vitreous humor, retina, choroid, sclera, and optic nerve. Figure 1 illustrates some unique anatomical barriers and features that protect the ocular microenvironment from infections and inflammation, making the eye an immunologically privileged organ.^4^ These barriers include:

(A) *The blood–retinal barrier (BRB):* formed by tight junctions between retinal capillary endothelial cells (inner BRB) or tight junctions between retinal pigment epithelial cells (outer BRB). The inner BRB mainly consists of the complex multicellular structure called the neurovascular unit, comprised retinal neurons, endothelial cells, pericytes, astrocyte foot processes, Müller cells, and microglia.^5^ The BRB is similar in function and structure to the blood–brain barrier, where it acts as a semiselective barrier that tightly regulates the movement of molecules, ions, and cells between the blood and the retina based on lipophilicity, molecular size, and charge.^6^^,^^7^ Although the small hydrophilic molecules follow the paracellular route through tight junctions, lipophilic molecules can cross the neurovascular unit via the transcellular route.^8^ The outer BRB consists of the retinal pigment epithelium (RPE), Bruch membrane, and the choroid. Tight junctions in the RPE prevent molecules and blood components from freely passing from the choriocapillaris blood vessels into the subretinal space. The Bruch membrane selectively facilitates the passive diffusion of small molecules, hindering the diffusion of large molecules.^9^ The tight control and selectivity of the inner and outer BRB contribute to preventing water, plasma components, and potentially toxic substances from entering the retina, thus preserving retinal microenvironment and homeostasis.^5^^,^^10^
(B) *The tear film barrier:* This is a transparent and thin fluid layer, around 3 *μ*m thick and 3 *μ*L in volume, that covers the outer surface of the eye with an osmolarity of approximately 300 mOsm. The tear film is composed of 3 layers: an outer lipid layer, an intermediate aqueous layer, and an inner mucin layer.^11^^,^^12^ The tear turnover rate is approximately 10% to 20%/min, with tear production ranging from 1 to 2 *μ*L/min.^13^ Ocular surface inflammation and irritation increase tear production to about 300 mL/min, diluting and rapidly eliminating administered drugs.^14^^,^^15^ This results in low intraocular drug bioavailability in the aqueous humor, with topical delivery often yielding only 0.1% to 5%.^16^ Tear turnover eliminates approximately 60% of drugs after 2 minutes of topical eye drop application. After 15 to 25 minutes, almost all the drug’s active ingredients are eliminated from the corneal surface.^17^
(C) *Corneal barrier:* The cornea is an avascular tissue composed of 5 distinct layers, namely the outer epithelium, Bowman membrane, intermediate stroma, Descemet membrane, and endothelium layer.^18^ The corneal epithelium is a protective barrier that shields the eye’s internal structures from foreign substances.^19^ The paracellular barrier of the corneal epithelium is primarily composed of tight junctions.^20^ The paracellular pores within the corneal epithelium measure approximately 1.6 nm in diameter, consequently limiting diffusion to molecules <500 Da.^21^^,^^22^ This, together with efflux transporters expressed in the corneal epithelium, limits the bioavailability of ocular therapeutics.
(D) *The vitreal barrier:* The vitreous humor is a homogeneous, viscoelastic hydrogel structure whose main component is water (>90%), along with solid fibrillar components such as glycosaminoglycans and collagen. The vitreous humor preserves the structural shape of the eye and provides support against the intraocular pressure. Hyaluronic acid is a glycosaminoglycan found in low concentrations in the vitreous, where it acts as a space-filling network between the collagen fibrils.^23^^,^^24^ Hyaluronic acid distribution is heterogeneous, and its concentration ranges from 65 to 400 *μ*g/mL in human vitreous.^23^ The net negative charge of the vitreous facilitates its selective charge-dependent diffusion, leading to the restriction of the diffusion of cationic particles.^25^^,^^26^

Despite these barriers’ protective role in the eye, they present considerable obstacles for ocular drug delivery. The dynamic barriers, such as the inner and outer BRB, lymphatic clearance, tear dilution, and efflux pumps, hinder drugs’ bioavailability and dosage maintenance.^27^

Few ocular drug administration strategies have emerged to overcome the ocular barriers and reduce regular clinical visits such as the port-delivery platform, hydrogels, and ocular inserts, where drug-eluting implants or a sustained-release device is inserted into the eye.^28^^,^^29^ These implantable and portable strategies provide sustained drug delivery over an extended period of time and can be used for the treatment of chronic eye conditions, such as glaucoma, DR, and AMD. The US Food and Drug Administration (FDA)–approved implant, Susvimo, uses a refillable port delivery system to replace frequent anti–vascular endothelial growth factor intravitreal injections with once or twice a year refills, thus improving patient compliance.^30^ However, these strategies carry risks such as device dislocation, infection, surgical complications, and enzymatic degradation, which may need further development to improve safety and efficacy.

Collectively, there is a need for developing targeted and efficient ocular drug delivery systems capable of addressing anatomical and physiological barriers in ocular tissue.

## 2 Advances nanosystem-based ocular drug delivery systems

Administering ocular pharmaceuticals poses considerable challenges because of the eye’s anatomical barriers that influence drug bioavailability. Several routes have been used for ocular drug administration.^31^, ^32^, ^33^ Figure 1 illustrates various ocular drug delivery routes, including topical, suprachoroidal, subretinal, peribulbar, retrobulbar, and intravitreal, combined with conventional systemic administration methods. Topical administration is the oldest route that constitutes 90% of the marketed formulation, with the advantages of self-administration and noninvasiveness.^34^ However, topical administration is challenged by the corneal barrier and tear dilution and turnover. Intravitreal administration offers direct delivery to the vitreous and retina with sustained drug levels but carries the risk of complications.^35^ The peribulbar and retrobulbar routes are routinely used to deliver local anesthetics around the eye, where there is debate over the safety and effectiveness of each route.^36^ Suprachoroidal administration provides elevated drug bioavailability within the choroid and retina, attributable to its proximity to the choroid.^35^^,^^37^ Subretinal administration into the subretinal space is considered an effective route for delivering ocular drugs to the photoreceptor layer and RPE.^38^

**Fig. 2:**
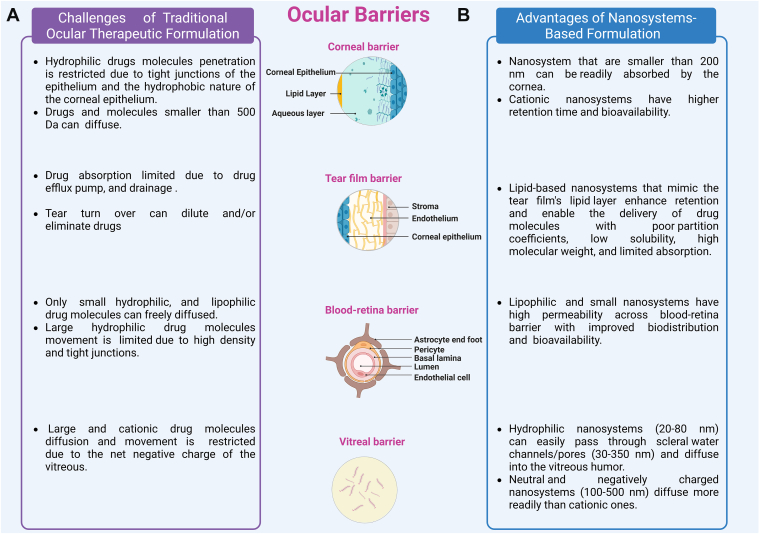
Ocular barriers challenge ocular drug delivery. (A) Traditional ocular drug formulations delivery and absorption are limited because of specific anatomical ocular obstacles. (B) Therapeutic formulations based on nanosystems. This approach, utilizing various strategies to overcome ocular barrier obstacles, improves ocular therapeutic absorption, retention, and bioavailability compared with traditional formulations.

A successful ocular drug carrier or delivery system must be small enough to bypass eye barriers and clearance mechanisms such as the BRB and tear film, while also being well tolerated. Nanosystem-based drug delivery uses materials at the nanoscale, usually between 50 and 400 nm, preferably under 100 nm, to package, transport, and release drugs. This approach can provide effective mucoadhesion and significantly overcome ocular barriers to reach the target site, improving stability and biodistribution profiles (Fig. 2).^39^^,^^40^ For instance, topical administration typically results in <5% of the total drug reaching the aqueous humor. However, nanosystems <200 nm are better absorbed by the cornea and conjunctiva following topical application.^41^^,^^42^ Given the negative charges of the cornea and the conjunctival surface, neutral or positively charged nanosystems have higher retention time.^43^ However, molecules bearing a positive charge tend to experience reduced permeability, because of their electrostatic interactions with the negatively charged proteoglycans within the scleral matrix.^44^ Transscleral penetration is limited by the scleral matrix of collagen fibrils.^45^ Utilizing hydrophilic NPs (20–80 nm) can easily pass through the scleral water channels/pores (30–350 nm in size), and diffuse into the vitreous humor.^46^^,^^47^ In the posterior segment, diffusion within the vitreous is a crucial factor influencing drug distribution in the vitreal cavity. The viscous and negatively charged vitreous humor can limit the distribution of macromolecule therapeutic agents and drugs. Cationic NPs (<139 nm) diffused approximately 10^3^ times more slowly within the porcine vitreous compared with anionic NPs (<170 nm). Their diffusion is only minimally impacted by vitreous liquefaction.^48^ Indeed, neutral and negatively charged nanosystems (100–500 nm in size) diffuse more readily than cationic nanosystems.^49^ However, nanosystem movement within the vitreous may be unpredictable and can lead to toxicity. In addition, aggregated and trapped NPs in ocular tissue may exhibit poor bioavailability and biodistribution. To overcome the issues of toxicity and aggregation, drug-encapsulated- or free NPs can be loaded in ocular drug delivery platforms such as hydrogels, and ocular inserts. These delivery systems protect NPs from ocular elimination or aggregation, and regulate drug release from the NPs.^50^ These nanocomposites have emerged as a promising delivery platform that combines the advantages of both systems. For example, researchers have encapsulated therapeutic molecules such as ranibizumab, sunitinib, and p11 antiangiogenic peptide within NPs-loaded hydrogels for the management of neovascularization in posterior segment eye pathologies.^51^, ^52^, ^53^ A comprehensive comparison of all NPs in nanocomposites models and nanosystems used in preclinical studies exceeds the scope of this paper, which focuses only on NPs as ocular drug delivery systems.

**Fig. 3:**
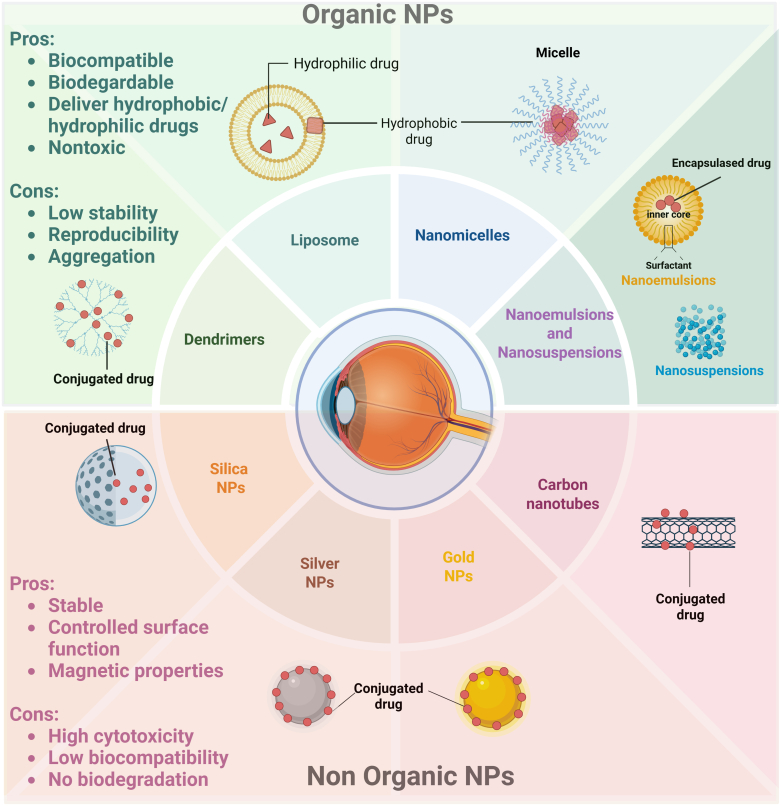
Illustration of the main nanosystems-based ocular drug delivery formulation for the treatment of ocular diseases. Organic materials used for NPs construction include lipids and polymers of either natural or synthetic origin. Organic NPs offer advantages in ocular drug delivery, including improved biocompatibility, biodegradability, and the ability to deliver both hydrophobic and hydrophilic drugs, and they are generally considered nontoxic. Organic NPs have some limitations regarding their low stability, low reproducibility, and systemic aggregation. Inorganic materials used for NPs construction include carbon, silica, and metals such as gold and silver. NPs within this class are exploited as therapeutic agents or nanosystems for ocular drug delivery because of their drug loading capacity and their unique physicochemical properties that enhance chemical stability and allow controlled surface functions along with useful magnetic properties. However, the application of inorganic NPs in ocular drug delivery has some limitations because of their cytotoxicity, low biocompatibility, and lack of biodegradation.

Nanosystem-based ocular drug delivery systems have emerged as a prominent focus within the domain of ophthalmic drug delivery research, in an attempt to surmount ocular barrier challenges, ultimately enhancing drug bioavailability, thus requiring lower doses to achieve therapeutic effects. Other potential advantages of nanosystems include sustained/controlled drug release, leading to reduced frequency of administration and improved patient compliance.^38^ Additionally, they hold future potential for ocular-targeted and gene delivery. Various nanomaterials with unique size-dependent physical and chemical properties are used, including organic and inorganic NPs, which are discussed in detail below. Organic NPs (based on carbon–hydrogen compounds) include liposomes, nanomicelles, nanosuspensions, nanoemulsions, dendrimers, and nanofibers. Inorganic NPs (based on metals and semiconductors) include gold NPs, silver NPs, silica NPs, and carbon nanotubes.^54^^,^^55^ Figure 3 illustrates various NPs in ocular drug treatments and their pros and cons.

### 2.1 Organic NP

Because of their nontoxic characteristics and biodegradability, organic NPs have a substantial advantage in ocular drug delivery. Commonly referred to as polymeric NPs, the organic NPs enumerated below exemplify some of the most critical types tested in ocular drug delivery.

#### 2.1.1 Liposomes

Liposomes are spherical lipid bilayers that closely resemble cellular membranes. Because of their specific composition and architecture, they demonstrate remarkable permeability and stability, facilitating their extensive utilization as carriers for hydrophobic and hydrophilic pharmaceutical agents with preferred diameters ranging from 50 to 10 nm.^56^^,^^57^ These lipid bilayers resemble the lipid layer that forms the tear film, allowing their use for dry eye treatment.^58^ Considering their ability to adhere to the cornea, liposomes are regarded as promising carriers for drugs characterized by a low partition coefficient, low solubility, high molecular weight, and limited absorption.^59^^,^^60^ In addition, the cationic lipids allow liposomes to adhere to the anionic mucin on the corneal epithelium, improving their retention. For instance, tacrolimus-loaded cationic liposomes have demonstrated efficacy in treating dry eye in preclinical models by enhancing ocular retention time and increasing the amount of tacrolimus gel in the cornea through interaction with the anionic ocular surface.^61^

Preclinical researchers applied various liposomal formulations for delivering several classes of drugs for ocular diseases, including antiglaucoma drugs,^62^, ^63^, ^64^, ^65^, ^66^, ^67^ anti-inflammatory drugs,^68^, ^69^, ^70^, ^71^ antibiotics,^72^, ^73^, ^74^, ^75^, ^76^ and drugs for ocular hypertension.^77^, ^78^, ^79^, ^80^, ^81^ These preclinical studies demonstrated that the liposomal formulation for ocular drug delivery effectively ameliorated various symptoms associated with ocular diseases, demonstrating significantly improved biocompatibility compared with marketed conventional formulations of the same drug. Approved liposomal formulations for clinical use include VISUDYNE for treating wet AMD and myopia, and Lacrisek for managing dry eye.^34^^,^^82^

#### 2.1.2 Nanomicelles and dendrimers

##### 2.1.2.1 Nanomicelles

Because of their unique spherical configuration, which possesses a hydrophilic shell and a hydrophobic core, nanomicelles are capable of effectively encapsulating hydrophobic pharmaceuticals within aqueous environments with improved solubility and bioavailability in the eye.^83^ Additionally, nanomicelles have high solubility and corneal penetration because of their small size (10–100 nm).^84^^,^^85^ However, further research is needed to ascertain the micelles’ intraocular biodistribution.

In relation to ocular diseases, preclinical research demonstrated that ocular delivery of hesperidin, a potent antibacterial agent, encapsulated into dipotassium glycyrrhizate micelles improved the drug’s therapeutic potential in rabbit models of ocular bacterial keratitis.^86^^,^^87^ This formulation exhibited good tolerance in rabbit eyes, improved in vitro passive permeation, and better ex vivo corneal permeation.^87^ It also improved hesperidin ocular bioavailability 5-fold as compared with a free hesperidin suspension solution.^87^

Micelle-delivered rapamycin exhibits enhanced therapeutic efficacy for experimental autoimmune uveitis in rats compared with rapamycin administration alone.^88^ For instance, treatment with rapamycin-loaded micelles was more effective at inhibiting the production of inflammatory cytokines in the vitreous humor of experimental autoimmune uveitis animals compared with treatment with rapamycin suspension.^89^ Additionally, methoxy polyethylene glycol-block-polycaprolactone micelles that were loaded with rapamycin exhibited prolonged and sustained drug retention within the experimental autoimmune uveitis rat retina up to 14 days post–intravitreal injection.^89^

##### 2.1.2.2 Dendrimers

These are a highly branched, three-dimensional structure resembling a tree or a star, comprising a central core and side chains with a size range of nearly 3–10 nm. Because of their unique structure, dendrimers exhibit high drug-loading efficiency, enabling them to deliver both lipophilic and hydrophilic medications through conjugation and encapsulation with no cytotoxicity. Iezzi et al. have demonstrated that the intravitreal administration of polyamidoamine dendrimers, which are loaded with the glucocorticoid fluocinolone acetonide can attenuate retinal inflammation and degeneration, with sustained release observed over a duration of 90 days in an animal model of retinal photoreceptor degeneration.^90^ Other preclinical studies have demonstrated that dendrimers can enhance ocular drug delivery for glaucoma,^91^, ^92^, ^93^ ocular infections,^94^ and AMD.^95^ Furthermore, dendrimers represent a promising strategy to improve the solubility of poorly soluble ocular pharmaceuticals.^96^^,^^97^ For instance, Molina-Martínez et al.^98^ developed water-soluble cationic and anionic carbosilane dendrimers as mucoadhesive polymers for the topical delivery of acetazolamide, which has poor aqueous solubility and ocular penetration. The authors exploit the high-affinity interaction between cationic carbosilane dendrimers and ocular transmembrane mucins and the tensioactive behavior observed for these polymers to improve the drug delivery. The topical administration of cationic carbosilane dendrimers containing acetazolamide improved its therapeutic efficacy, indicated by a rapid (within 1 hour) and extended (up to 7 hours) hypotensive effect.^98^ However, cytotoxicity has been reported in cationic dendrimers with a high density of positive charges.^99^ Molina-Martínez et al demonstrated that their carbosilane dendrimer was well tolerated when used in a safe concentration range of 5 to 10 *μ*M.^98^

#### 2.1.3 Nanoemulsions and nanosuspensions

Nanoemulsions represent the most extensively researched and widely used within nanotechnology drug delivery systems, specifically in ocular local drug delivery. Owing to their distinctive characteristics, which encompass a diminutive size of <200 nm and an elevated surface contact area, these substances emerge as potential drug solubilizers and stabilizers.^100^ Nanoemulsion-based drug delivery’s ability to cross the corneal tight junctions improves the drug bioavailability and residence time compared with conventional methods, resulting in a significant reduction in side effects associated with frequent administration of large drug doses using conventional methods.^101^ Nanoemulsions also facilitate the sustained release of drugs to promote efficient absorption by the cornea, sclera, or conjunctiva.^102^ Therefore, Nanoemulsions are the first choice for treating many eye diseases, such as infection, dry eye disease, glaucoma, and inflammation.^103^, ^104^, ^105^, ^106^ For instance, Taayel et al. evaluated the pharmacokinetics and bioavailability of topically administered Terbinafine hydrochloride-loaded nanoemulsion gel in the aqueous humor of rabbits.^106^ The authors demonstrated that nanoemulsion significantly increased the maximum concentration, delayed time to peak maximum concentration, prolonged mean residence time, and increased bioavailability by 2-fold compared with the oily drug solution form.^106^

Nevertheless, nanoemulsions exhibit certain limitations in ocular drug delivery, including ocular irritation and blurred vision attributable to elevated surfactant concentrations and increased viscosity. In addition, the physicochemical properties of nanoemulsions can lead to their degradation over time.^107^

Nanosuspensions represent a distinctive category of drug delivery systems that do not necessitate carrier materials, comprised solely of pure submicron-sized (<1 *μ*m) pharmaceutical active ingredient particles, which are frequently stabilized by surfactants or polymers. The nanoscale particle size effectively resolves the issue of low solubility, augmenting the surface area for contact and enhancing both the duration and bioavailability of pharmaceutical agents within the cornea and ocular tissue.^108^ Previous preclinical studies have conclusively demonstrated that nanosuspensions are effective in delivering various pharmaceutical compounds, including hydrocortisone,^109^ cyclosporine,^110^ pranoprofen,^111^ triamcinolone acetonide,^112^ and moxifloxacin,^113^ to ocular tissues with an enhanced retention time compared with the respective drugs alone.

For example, Yan et al^114^ engineered cationic and drug-focused mucus-penetrating particles nanosuspensions to deliver cyclosporine A to the anterior ocular tissues via topical drop instillation. The authors evaluated mucoadhesion between the porcine mucin and the engineered cationic nanosuspensions, and showed that cationic nanosuspensions enhanced the interaction by 5- to 6-fold compared with drug-focused mucus-penetrating particles nanosuspensions. Porcine mucin was significantly stronger compared with drug-focused mucus-penetrating particles nanosuspensions. In addition, both nanosuspension formulations successfully delivered cyclosporine A to the anterior eye tissue at therapeutic levels and improved bioavailability.^114^ Another in vivo study showed that loading diclofenac/chitosan in methoxy polyethylene glycol-block-polycaprolactone cationic nanosuspension significantly improved the pharmacokinetic parameters. For instance, the methoxy polyethylene glycol-block-polycaprolactone cationic nanosuspension had enhanced corneal retention time, permeation, and bioavailability (2.3 times higher) of diclofenac compared with the commercial diclofenac eye drops. However, the cationic nanosuspension formulation caused a temporary ocular irritation that resolved after 24 hours of instillation with no indication of cytotoxicity.^115^ A recent in vivo preclinical study demonstrated that the moxifloxacin-loaded nanosuspension had around 2.6-fold longer half-life in the cornea compared with commercial moxifloxacin drops (Vigamox–antibiotic), with a safe profile.^116^

#### 2.1.4 Organic nanofibers

As stated by the National Science Foundation, nanofibers are defined as solid fibers possessing diameters ranging from 50 to 500 nm, with at least one dimension measuring ≤100 nm.^117^ The efficacy of utilizing nanofibers for ocular drug delivery varies and depends on the type of scaffold polymer, its molecular weight, and the hydrophobicity/hydrophilicity characteristics of the drug.^118^ However, the drug release is highly dependent on the diameter of nanofibers. The exceptionally high surface area of the nanofibers allows high loading drug capacity in a very small area. This advantage renders these entities powerful tools for developing advanced drug delivery systems with diverse biomedical applications for ocular diseases. For instance, Angkawinitwong et al^119^ have used electrospun nanofiber formulations for the intraocular sustained release of bevacizumab for the management of AMD experimentally, which exhibited half-lives of 11 to 14 days. Mirzaeei et al^120^ used a topical modified nanofiber to sustain the release of the antibiotic, ofloxacin, in the ocular tissue of an in vivo animal model. Also, nanofibers have been used for local ocular delivery of cyclosporine for immunosuppression,^121^ and timolol maleate for managing intraocular pressure.^122^

### 2.2 Inorganic NPs

#### 2.2.1 Gold NPs

Gold NPs are recognized for their chemical durability, capacity for modification, and biocompatibility.^123^^,^^124^ The spherical and cubic formulations, characterized by dimensions ranging from 50 to 100 nm, have exhibited a sustained biocompatible and safe profile in retinal pigment epithelial cells,^125^ thereby facilitating their application for ocular drug delivery. Wang et al^126^ have successfully encapsulated and stabilized the cataract treatment, N-acetylcarnosine in NPs. Furthermore, preclinical studies have demonstrated that gold NPs effectively encapsulate drugs for treating glaucoma and dry eye syndrome.^127^^,^^128^

Previously, Söderstjerna et al^129^ evaluated the toxicity of gold NPs on the mouse retina. The authors showed that oxidative stress and apoptosis were induced in retinal cells at low concentrations of gold NPs (0.4 *μ*g/mL 80 nm NPs and 0.0065 *μ*g/mL 20 nm NPs). Additionally, gold NPs caused glial activation and photoreceptor toxicity. Karakoçak et al^125^ conducted an in vitro evaluation of gold NPs and showed that the toxicity of gold NPs on RPE cells is highly dependent on their shape, size, concentration, and surface area.

Conversely, Song et al^130^ demonstrated no effects on retinal integrity and function after intravitreal injections of 160-nm–sized gold nanodisks at a concentration of 10 pm in mice. This inconsistency could be due to: (1) different models used; (2) the functionalization of gold NPs and their positive surface charge; and (3) the particles’ size, shape, and concentration. These findings indicate that NPs’ characteristics substantially affect their toxicity.

#### 2.2.2 Silver NPs

NPs composed of silver are used as a therapeutic vehicle owing to their established antimicrobial, antioxidant, and antiangiogenic properties.^131^, ^132^, ^133^ The antiangiogenic properties are ascribed to inhibiting the hypoxia-inducible factor 1*α* within cells, subsequently suppressing the vascular endothelial growth factor A function.^134^ However, the study showed that silver NPs at 50 *μ*g/mL notably decreased, whereas 200 *μ*g/mL entirely prevented the accumulation of hypoxia-inducible factor 1*α*, which led to cytotoxicity. Silver NPs have been documented to induce ocular toxicity in animals, which may restrict their application in ocular drug delivery.^134^^,^^135^ For instance, Quan et al^136^ evaluated the cytotoxicity of silver NPs on human RPE cells in vitro at concentrations of 0.2, 1, 5, 25, and 125 *μ*g/mL for 24 hours. The authors showed that silver NPs induced RPE mitochondrial apoptosis in a dose-dependent manner.^136^

#### 2.2.3 Silica NPs

Because of their biocompatibility, extensive specific surface area, and ease of fabrication, mesoporous silica NPs, with pores ranging from 2 to 50 nm in diameter, are the most extensively researched inorganic NPs for ocular drug delivery. Additionally, silica NPs are biodegradable and possess a large drug loading capacity.^137^^,^^138^

It was demonstrated that injecting silica NPs into the vitreous cavity effectively reduced abnormal retinal angiogenesis in mice with oxygen-induced retinopathy, suggesting that they have an antiangiogenic effect similar to silver NPs.^139^ Silica NPs provide a practical tool for drug delivery to manage ocular diseases. For instance, Liao et al^140^ used modified silica NPs to sustain the release of pilocarpine for glaucoma treatment in the anterior chamber after intracameral injection up to 21 days. Kim et al^141^ have used amino-functionalized mesoporous silica particles for the ocular delivery of brimonidine for glaucoma. The authors demonstrated that their engineered mesoporous silica particles exhibited high mucoadhesive properties because of the presence of hydroxyl and amino groups on the surface, allowing for hydrogen bonds and an ionic complex with the mucin.

#### 2.2.4 Carbon nanotubes

Carbon nanotubes (CNTs) lack the carbon–hydrogen bonds characteristic of organic NPs and, therefore, are considered inorganic. Because of their substantial surface area, multifunctional surface chemistry, biocompatibility, and distinctive needle-like structures that can readily penetrate cell membranes, CNTs have garnered considerable interest within the biomedical field, particularly in the discipline of ophthalmology, owing to their potential applications in ocular drug delivery.^142^, ^143^, ^144^ Previous reports demonstrated the durability and effective controlled release of CNTs for ocular drug delivery in preclinical models of DR, AMD, and glaucoma.^145^^,^^146^ In addition, Demirci et al^147^ demonstrated that CNTs labeled with fluorescein isothiocyanate, targeting ligands biotin (CNT-fluorescein isothiocyanate-biotin) and folic acid (CNT-fluorescein isothiocyanate-folic acid) successfully penetrated the retinoblastoma tumor in mice eyes, strongly suggesting the imaging (diagnostics) and therapeutic potential of CNTs. Eleftheriou et al^148^ evaluated the role of CNT as promising materials for epiretinal prosthetic devices and evaluated their interactions with retinal ganglion cells in vivo and ex vivo. The authors showed that the structural changes of CNT over time lead to functional alteration in CNT electrodes and their ability to stimulate retinal ganglion cells.^148^ Given these findings, effective and sustained drug delivery highly depends on CNT-based formulation and stability, where it should maintain its structure without aggregation or degradation.^149^

In another cytotoxicity evaluation of CNT, Yan et al^150^ demonstrated that single-walled CNT reduced cell survival, changed reactive oxygen species levels, compromised membrane integrity, and induced apoptosis, which is very toxic to human RPE cells in vitro. However, functionalization of CNT with hydroxy and carboxylic acid groups enhanced their biocompatibility.^150^

## 3 Clinical applications

The unique properties of these nanoystems and the ongoing development of innovative formulations make them a suitable candidate for drug delivery to the eye. Utilizing nanosystems for ocular drug delivery provides several benefits, such as the ability to encapsulate and deliver both hydrophilic and hydrophobic drugs, minimizing potential systemic side effects, and improving therapeutic efficacy through controlled and sustained release, which results in reduced drug dosage. Table 1 summarizes some clinical trials that used nanosystems for ocular drug delivery.

**Table 1:** Summary of the current status of human clinical trials utilizing nanosystems for ocular drug delivery Total number of clinical trials: nanoemulsions, 8; liposomes, 17; micelles, 3; dendrimers, 2; trimetallic nanoparticles, 1; albumin microspheres, 1; magnetic nanoparticles, 1; and not available for formulation, 1.

	Trial Number and Title	Condition	Interventions	Phases	Status	Outcomes
Nanoparticles	NCT05343156: Efficacy and Safety of Dexamethasone Nanoparticles Eye Drops in Diabetic Macular Edema	DME	Dexamethasone	II	Completed	144 patients received OCS-01 thrice daily for 12 wk. In the treatment group, 91 of 99 patients saw reduced central retinal thickness compared with placebo (−53.7 *μ*m vs −16.9 *μ*m, *P* = .0115). The drug was safe overall, with a higher incidence of increased IOP in the treatment group.
	NCT01523314: Topical Dexamethasone – Cyclodextrin Microparticle Eye Drops for Diabetic Macular Edema	DME	Dexamethasone	II/III	Unknown	NA
	NCT03001466: A Randomized Controlled Trial Comparing Urea Loaded Nanoparticles to Placebo: A New Concept for Cataract Management	Cataract	Urea	II	Completed	NA
	NCT00738361: Paclitaxel Albumin-Stabilized Nanoparticle Formulation in Treating Patients With Metastatic Melanoma of the Eye That Cannot Be Removed by Surgery	Intraocular melanoma	Nab-paclitaxel	II	Completed	Only 4 patients were included in the study. Median progression-free survival: 6.2 (1.7–13.0).
	NCT06181227A: Phase 2 Study of Intravitreal AVD-104 in Diabetic Macular Edema	DME/DR	AVD-104	II	Terminated	The trial was discontinued because of PI supply issues and study funding constraints.
	NCT04130802: OCS-01 in Treating Inflammation and Pain in Post-cataract Patients	Postoperative pain	OCS-01 (dexamethasone cyclodextrin)	II	Completed	On day 4, 72.5% of eyes had no pain with OCS-01 once daily, 62.7% with OCS-01 twice daily, and 45.1% with the vehicle. Statistical significance was observed for OCS-01 once daily (*P* = .005) and OCS-01 twice daily (*P* = .074) compared with the vehicle. OCS-01 was well tolerated.
	NCT04894110: Study of Safety and Tolerability of EO2002 in the Treatment of Corneal Edema	Corneal edema	EO2002	I	Completed	NA
Liposomes	NCT03617315: Crosslinked Hyaluronic Acid With Liposomes and Crocin in Dry Eye	Dry eye	Hyaluronic acid	NA	Completed	NA
	NCT04115800: Liposomal Sirolimus in Dry Eye Disease	Dry eye	Liposomal sirolimus	Early I	Completed	NA
	NCT02992392: Comparison of the Clinical Effects of Two Tear Substitutes in Patients With Dry Eye Syndrome	Dry eye	Liposic	NA	Unknown	NA
	NCT01987323: Safety and Efficacy of Liposomal Latanoprost in Ocular Hypertension	Ocular hypertension	Liposomal latanoprost	I/II	Completed	
	NCT06478134: Efficacy and Safety of a Multiple-Action Tear Substitute (TriMix) in Dry Eye Disease	Dry eye	Trimix tear substitutes	II/III	Completed	NA
	NCT04087733: Effectiveness of Liposomal Ozonized-Oil for Cataract Surgery	Ocular infections	OZODROP	III	Completed	NA
	NCT00535054: Study to Assess the Safety and Patients’ Satisfaction of Tears Again∗ in the Treatment of Dry Eye Symptoms	Dry eye	Tears Again	NA	Completed	NA
	NCT03211351: Effects of Two Tear Substitutes in Patients With Dry Eye Syndrome	Dry eye	Liposic	NA	Completed	NA
	NCT05551598: Efficacy and Safety of Mitoxantrone Hydrochloride Liposome	Neuromyelitis	Mitoxantrone hydrochloride liposome	II	Completed	NA
	NCT00506142: Safety and Efficacy of Marqibo in Metastatic Malignant Uveal Melanoma	Uveal melanoma	MarqiboÂ (vincristine sulfate liposomes injection)	II	Completed	Marquibo (vincristine sulfate liposome injection) demonstrated acceptable tolerability in melanoma patients with hepatic impairment.
	NCT02466399: POLAT-001 Compared to Latanoprost Ophthalmic Solution	Glaucoma	POLAT-001	II	Completed	At month 3, treatment with subconjunctival liposomal latanoprost resulted in a mean reduction in IOP of −2.3 mm Hg (SD, 4.6), compared with −6.4 mm Hg (SD, 2.9) observed with latanoprost ophthalmic solution. Adverse events were reported exclusively in the liposomal latanoprost group, with the most frequent events being conjunctival hemorrhage (26.4%), foreign body sensation (17.0%), and conjunctival hyperemia (13.2%).
	NCT00072384: Systemic Chemotherapy and Subtenon Carboplatin, and Local Ophthalmic Therapy in Children With Intraocular Retinoblastoma	RB	Liposomal vincristine sulfate	III	Completed	Patients who received 6 courses of liposomal vincristine sulfate intravenous injection (IV), carboplatin IV, and etoposide in group C eye had a probability of treatment failure of 0.25 (0.217); patients who received the same courses of treatment in group D eye showed a probability of treatment failure of 0.52 (0.102299).
	NCT00335738: Vincristine, Carboplatin, and Etoposide or Observation Only in Treating Patients Who Have Undergone Surgery for Newly Diagnosed Retinoblastoma	RB	Liposomal vincristine sulfate	III	Completed	Patients who received 6 courses of liposomal vincristine sulfate IV, carboplatin IV, and etoposide had a probability of EFS of 0.9628 (0.8890–0.9878); patients who underwent observation periodically (identified by central review as not high risk) for at least 5 y showed a probability EFS of 1 (1 to 1).
	NCT03093701: TLC399 (ProDex)	CRVO	TLC399 (ProDex)	II	Completed	TLC399 (ProDex) treatment in patients with macular edema due to RVO lead to gain of at least 15 letters in BCVA from baseline after 12 mo of treatment.
	NCT02006147: Phase 1 Open-label Study to Evaluate Efficacy and Tolerability of TLC399	CRVO/BRVO	TLC399	I/II	Completed	TLC399 was well tolerated in patients with macular edema caused by RVO, without dose-limiting toxicities, while demonstrating efficacy improvements in both the reduction of retinal central subfield thickness and visual acuity.
	NCT04018417: Evaluation of Amphotericin B in Optisol-GS for Prevention of Post-Keratoplasty Fungal Infections	Fuch dystrophy	Amphotericin B	II/III	Withdrawn	NA
Dendrimers	NCT05105607: A Study to Evaluate the Safety, Tolerability, and Pharmacokinetics of D-4517.2	AMD/DME	D-4517.2	I	Completed	NA
	NCT05387837: Safety, Tolerability and PK of Subcutaneous D-4517.2	AMD/DME	D-4517.2	II	Active/not recruiting	NA
Micelles	NCT01576952: Comparison Study of ISV-303 to DuraSite Vehicle	Inflammation	ISV-303	III	Completed	NA
	NCT03192137: Study to Evaluate ISV-305 Compared to Vehicle	Inflammation	ISV-305	III	Completed	ISV-305 treatment, a combination of dexamethasone and crosslinked polyacrylic acid and chitosan polymers, improved the therapeutic efficacy in alleviating inflammation and pain post–ocular surgery.
	NCT06188260: Efficacy of a New Nanoemulsion Artificial Tear	Dry eye	Systane COMPLETE Lubricant Eye Drops	II	Completed	NA
	NCT03785340: Study of Brimonidine Tartrate Nanoemulsion Eye Drop Solution	Dry eye	Brimonidine Tartrate	III	Completed	Significant reduction in dry eye symptoms as measured by the Symptom Assessment in Dry Eye (SANDE) questionnaire as early as 28 days, with safe profile.
	NCT03591874: Study of Brimonidine Tartrate Nanoemulsion Eye Drops	Ocular surface disease	Brimonidine Tartrate for treatment of oGVHD	III	Terminated	Brimonidine nanoemulsion effectively relieved ocular surface inflammation and dryness in oGVHD patients with a favorable safety profile.
	NCT05724446: Clobetasol Propionate Ophthalmic Nanoemulsion, 0.05% in the Treatment of Inflammation After Cataract Surgery in Pediatric Population	Cataract	Clobetasol propionate	III	Recruiting	NA
	NCT04246801: Clobetasol Propionate Ophthalmic Nanoemulsion 0.05% With Cataract Surgery (CLOSE-1)	Cataract	Clobetasol propionate	III	Completed	In the clobetasol treatment group, 37.8% of patients had complete absence of cells in the anterior chamber by day 8, compared with 18.1% in the placebo group.
	NCT04249076: Clobetasol Propionate Ophthalmic Nanoemulsion 0.05%	Cataract	Clobetasol propionate	III	Completed	In the clobetasol treatment group, 27.3% of patients had complete absence of cells in the anterior chamber by day 8, compared with 9.9 % in the placebo group.
	NCT03492541: Study of Efficacy and Tolerability of SYSTANE Complete	Dry eye	Propylene glycol-based eye drops	NA	Completed	NA
	NCT04631315: Difluprednate vs a Prednisolone Acetate – Phenylephrine on Postoperative Inflammation	Cataract	Difluprednate ophthalmic emulsion 0.05%	IIII	Completed	NA

BRVO, branch retinal vein occlusion; CRVO, central retinal vein occlusion; DME, diabetic macular edema; EFS, events-free survival; IOP, intraocular pressure; IV, intravenous injection; NA, not available; PI, principal investigator; RB, retinoblastoma; RVO, retinal vein occlusion; BCVA, best corrected visual acuity.

Examples of successful FDA-approved nanosystems applications in ocular drug delivery include Restasis, a nanoemulsion, and Cequa, which uses nanomicellar technology. Both formulations are used to deliver high concentrations of cyclosporine A for dry eye treatment.^55^ A meta-analysis of 4107 participants found that Restasis was among the most effective treatments compared with placebo on several subjective and objective measures of effectiveness. However, Cequa was excluded from this meta-analysis because the clinical trials’ outcomes measured only the number of people who improved.^151^ During the clinical trial, Cequa met the endpoint at day 84 and showed clinically meaningful improvement for both eyes, increased tear production, as well as a substantially greater change from baseline in total corneal fluorescein staining at all common postbaseline time points (day 28, 56, and 84; *P* ≤ .0013).^152^, ^153^, ^154^

Additionally, several other drugs that use nanosystems for drug delivery have been FDA-approved for the treatment of ocular disease, such as dry eye, postoperative pain and inflammation, bacterial infections, conjunctivitis, and glaucoma.^55^ Other successful examples of commercial nanosystems in ocular drug delivery include Cyclokat,^155^ which is a nanoemulsion of cyclosporine A; Lacrisek, a liposomal formulation of vitamin A palmitate and vitamin E; Artelac Rebalance, a liposomal vitamin B12; and Ikervis, a nanoemulsion used for treating acute keratitis in dry eye syndrome.^156^ However, there is a lack of clinical trials examining the long-term safety, and potential accumulation and aggregation of nanoparticles in the eye, highlighting the need for future clinical studies to confirm their safety for chronic use.

The prospects for advancements in nanosystem technology for the delivery of pharmaceuticals targeting ocular diseases seem to be increasingly promising and essential.

In addition to the enhanced therapeutic efficacy, cost-benefit analysis on nanosystems at scale will demonstrate their economic feasibility. For example, Suriyaprakash et al^157^ conducted a preliminary cost analysis on dorzolamide nanoparticles compared with commercial dorzolamide eye drops for glaucoma treatment. The authors demonstrated that dorzolamide nanoparticles are economically feasible to manufacture on a large scale. Further cost-benefit analyses are needed for promising nanosystems to ensure their successful application in the clinical setting.^157^

## 4 Challenges

Despite significant and ongoing advancements in the development and use of nanosystems together with an increasing number of successful preclinical studies and promising clinical trials, the future clinical applications of these systems still face challenges. Notable obstacles include:

(1) Discrepancy between preclinical and clinical results: Selected factors that may contribute to this discrepancy include:
  (i) The inconsistency between in vivo and in vitro results. Although cell lines can facilitate identification of direct therapeutic mechanisms, they lack the in vivo models’ geometric structure and cellular complexity.
  (ii) The discrepancies between animal models and human diseases.^158^ Preclinical studies have shown successful topical delivery of small molecules or proteins to the retina in rodent models. However, caution is needed when interpreting efficacy data because anatomical differences between rodent and human eyes can influence pharmacokinetics, drug metabolism, and long-term ocular tolerance. For instance, in humans, the sclera is thinner and more permeable to some drugs, which enables easier penetration into the eye and access to target tissues.^159^ Therefore, larger species such as rabbits, dogs, pigs, or monkeys should be used to test new nanosystems for ocular drug delivery strategies to increase their likelihood of successful translation to the clinic.^159^
  (iii) Lack of a uniform ocular drug distribution by the nanosystem. Exploring the pharmacokinetics of nanosystems in the eye is crucial to understanding their absorption, distribution, metabolism, and how they interact with ocular tissues. To address these challenges, it is essential to pay particular attention to the nanosystem interaction with the immune system, biocompatibility, and biodistribution, which are influenced by surface coatings, targeting moieties, and the shape and size of the nanosystems.
  (iv) The inconsistency and occasional conflicting preclinical data arising from diverse fabrication methods of inorganic nanosystems, resulting in differences in their size and surface charge. NPs materials characterization and/or validation methods lack international standardization. Therefore, there is a need to develop consensus standards to improve the efficient development and regulatory review of NPs-containing drug products. In this context, creating standardization bodies focusing on NPs-containing drugs and/or medical devices would be highly desirable and could constitute the international framework for NPs fabrication methods, validation protocols, and uniform terminology.
(2) Industrial-scale production: Large-scale production of these delivery nanosystems is complex and presents several challenges. Precise control over the nanosystems’ production processes, such as mixing, extrusion, homogenization, evaporation, centrifugation, lyophilization, or sterilization, is required to maintain consistent nanosystems characteristics as well as uniformity among batches. Additionally, high-scale production increases production costs, representing economic challenges that require innovative approaches.^160^^,^^161^ These challenges demand sophisticated manufacturing methods to speed up the effective use of nanosystems in ocular drug delivery.

## 5 Ethical and regulatory aspects

Regulatory hurdles arising from nanomaterials’ emergent and intricate nature contribute to the absence of definitive protocols for demonstrating safety, efficacy, and manufacturing consistency. Regulatory agencies frequently lack international harmonization and standardized definitions and terminology concerning nanomedicines, especially the application of NPs. Understanding guidelines from regulatory bodies such as the FDA and the European Medicines Agency is essential to accelerate the complex translation process. For example, although both the European Medicines Agency and FDA recognize the 1 to 100 nm range for a nanomaterial, the FDA may consider a material a nanomaterial if it is engineered to have specific properties and exhibits these characteristics, even if one dimension is >100 nm, ≤1000 nm.^162^^,^^163^ Given the inherent complexity of these products, applications for marketing authorization of future nanosystems are expected to entail prolonged approval timelines, which require comprehensive data regarding their pharmacokinetic, biocompatibility, and stability profiles under various physiological and pathological conditions. Another significant challenge is the absence of standardized in vitro and in vivo protocols for assessing the cytotoxicity of nanosystems across different ocular tissues, which is essential for accurate safety evaluations. Owing to their improved permeability in ocular tissue, systemic aggregation of nanosystems that may cause toxicity in different retinal layers is a significant risk and concern.^164^

There is a lack of standardized safety and preclinical assessment of NPs before drug conjugation. The current safety assessment, which follows the guidelines of the International Council for Harmonisation and the International Organization for Standardization, applies to medicinal products and medical devices, respectively.^165^ Currently, only a few standardized methods are available to assess the biological effects of nanotechnology-based products. The International Organization for Standardization guidance on biological evaluation of medical devices features a dedicated section for nanomaterial-containing products (ISO/TR 10993–22:2017). However, this section emphasizes characterizing the pharmacokinetic profiles of nanomedicinal products, which can differ markedly from NPs and/or NPs-containing drugs. Therefore, there is a need for appropriate standardized methods to supplement existing safety testing protocols outlined by International Council for Harmonisation guidelines, or initiating a separate international standardization forum that covers that specific area of NPs and/or NPS-containing drugs.^166^

Additionally, nanosystems can influence how a drug interacts with proteins and cellular structures, as well as its distribution and accumulation within tissues and organs. In contrast, traditional pharmaceutical formulations primarily affect bioavailability by regulating the drug release. Therefore, evaluating bioavailability through drug profiles in the bloodstream must differ between nanotech-based and conventional forms, posing a significant challenge in preclinical and clinical evaluations.^167^ In this context, the FDA and European Medicines Agency have issued guidelines for various nanomedicines (such as liposomes, iron-core particles, and block-copolymer micelles) to outline the additional data needed for characterizing first-in-human products or comparing subsequent ones.^168^

## 6 Future directions

Having discussed the major challenges, we next turn to future directions to achieve the successful application of nanosystems in ocular drug delivery and therapy. For example, nanosystems-mediated gene therapy and RNA-based therapies present an alternative to conventional viral vectors in the treatment of ocular genetic diseases. Herrera-Barrera et al^169^ have demonstrated that peptide-conjugated lipid NPs can facilitate the delivery of mRNA to the neural retina in nonhuman primates after subretinal injection, suggesting the utility of lipid NPs-mRNA therapies for inherited blindness. Apaolaza et al^170^ have demonstrated successful delivery of a therapeutic plasmid that encodes the protein retinoschisin in vitro, utilizing solid lipid NPs-based vectors. These lipid-based NPs are similar to micelles, but are distinguished by featuring a solid lipid core. This suggests a novel method for the treatment of X-linked juvenile retinoschisis using nanosystem-based gene therapy.^170^ This sophisticated approach of nanosystems-based gene therapy offers a targeted, sustained release, and specific delivery of genetic materials, which minimizes systemic exposure and potential side effects.

Modified dendrimers exhibit more favorable pharmacological profiles in the nanosystem compared with conventional dendrimers and bear translational opportunities in dendrimer-based ocular drug delivery.^171^ Modified dendrimers exhibited improved pharmacokinetics, with greater specificity to the target site, rapid intracellular uptake, higher bioavailability, longer blood circulation, and enhanced in vivo stability. However, regulatory barriers due to chemistry, manufacturing, and control standards limit the clinical application and commercialization of modified dendrimers. To overcome these limitations, a pre–good manufacturing practice method should be used for the verification characterization step to verify the biophysical characteristics of NPs and ensure precision between different instruments and sample lots. In addition, determining the *ζ* potential of colloidal nanoparticles at a range of specific pH measurements, and testing under ocular-mimicking conditions is recommended to determine the isoelectric point and the NPs stability condition.

In addition, structural limitations due to an increase in size with the newer advancement of generations pose an additional barrier to successful clinical application. To overcome these limitations, initiating strong collaborations that bring regulatory bodies, formulation scientists, and clinicians together is highly advisable to achieve advancements in the application of modified dendrimers for ocular drug delivery.

In addition to modified dendrimers, translational research on CNTs requires a deeper understanding of their interactions and immunogenicity at both cellular and molecular levels to achieve therapeutic durability and biocompatibility for ocular drug delivery. This knowledge is crucial for predicting the long-term effects of CNTs and ensuring ocular safety.

Smart drug-delivery nanosystems are another major future avenue in the field of ocular drug delivery. For instance, designing nanosystems that disrupt their structure in response to external stimuli allows for targeted and premature drug release. Future investigations should explore and develop smart drug-delivery nanosystems using biocompatible and nontoxic materials. In addition to smart drug-delivery nanosystems, mucoadhesive/mucopenetrative nanosystem formulations present a promising field for innovative development for ocular drug delivery. Despite their improved sustained release and ocular retention, future studies are warranted to overcome their limited stability and shelf-life.

Nanosystem-based drug delivery shows excellent potential for ocular conditions with significant unmet therapeutic needs, such as posterior uveitis, macular edema, DR and AMD.^172^ These conditions often require invasive treatments such as intravitreal injections and have variable patient responses because of age, genetic predisposition, disease stage, and systemic comorbidities.^172^ These areas represent high-priority targets for the translation of nanosystem-based ocular therapeutics.

Finally, the application of nanosystems in personalized medicine offers enhanced targeting capabilities, facilitating significantly higher bioavailability compared with conventional drugs at the than the maximum tolerated doses, which are often linked to adverse off-target effects. Consequently, the dosage can be customized according to each patient’s specific medical condition.^173^^,^^174^

## 7 Conclusion

Overall, the application of nanosystems in ocular drug delivery has the potential to significantly impact human health. It presents encouraging prospects for focused, efficient, and personalized treatments aimed at enhancing outcomes for eye diseases. Notwithstanding their significant potential, innovative nanosystems face numerous obstacles, including inconsistent ocular drug distribution, complex approval processes from regulatory bodies, and escalating concerns regarding nanotoxicity across various ocular layers. These challenges urge the need for interdisciplinary collaboration among researchers, clinicians, and industry professionals to expedite innovation and overcome barriers in translating promising preclinical results into effective clinical ocular therapies, ultimately enhancing patient health.

## Conflict of interest

The authors declare no conflicts of interest.
